# Surgery or Endoprosthesis for Malignant Obstructive Jaundice

**DOI:** 10.1155/1989/61568

**Published:** 1989

**Authors:** Jeffrey B. Matthews

**Affiliations:** Department of Visceral and Transplantation Surgery University of Berne, Inselspital 3010 Berne Switzerland


in a clinical investigation would be to include only potential transplant candidates at the outset. Ideally, such a study would be randomized with one arm including patients who either underwent initial hepatic transplantation or transplantation after sclerotherapy failure and the second arm consisting of individuals who would receive a shunt operation after sclerotherapy if their hepatic functional reserve was well maintained. This group would then undergo transplantation as a back-up to shunt surgery when end-stage liver disease was apparent. Because many variceal bleeders are not candidates for hepatic transplantation, it would be difficult to accrue a sufficient number of patients to such an investigation to provide meaningful results.

Layton F. Rikkers, M.D.
Professor and Chairman, Department of Surgery
University of Nebraska Medical Center
42nd and Dewey Avenue
Omaha, Nebraska 68105

### REFERENCES

1. Iwatsuki, S., Starzl, T.E., Todo, S., Gordon, R.D., Tzakis, A.G., Marsh, J.W., Makowka, L., Koneru, B., Stieber, A., Klintmalm, G., Husberg, B., van Thiel, D. (1988) Liver transplantation in the treatment of bleeding esophageal varices. *Surgery*; **104**: 697–705.
2. Starzl, T.E., van Thiel, D., Tzakis, A.G., Iwatsuki, S., Todo, S., Marsh, J.W., Koneru, B., Staschak, S., Stieber, A., Gordon, R.D. (1988) Orthotopic liver transplantation for alcoholic cirrhosis. *JAMA*; **260**: 2542–2544.
3. Warren, W.D., Henderson, J.M., Millikan, W.J., Galambos, J.T., Brooks, W.S., Riepe, S.P., Salam, A.A., Kutner, M.H. (1986) Distal splenorenal shunt versus endoscopic slcerotherapy for long-term management of variceal bleeding. *Ann. Surg*; **203**: 454–462.
4. Rikkers, L.F., Burnett, D.A., Volentine, G.D., Buchi, K.N., Cormier, R.A. (1987) Shunt surgery versus endoscopic sclerotherapy for long-term treatment of variceal bleeding. *Ann. Surg*; **206**: 261–271.
5. Teres, J., Bordas, J.M., Bravo, D., Visa, J., Grande, L., Garcia-Valdecasas, J.C., Pera, C., Rodes, J. (1987) Sclerotherapy versus distal splenorenal shunt in the elective treatment of variceal hemorrhage: a randomized controlled trial. *Hepatology*; **7**: 430–436.
6. Brems, J.J., Hiatt, J.R., Klein, A.S., Millis, J.M., Colonna, J.O., Quinones-Baldrich, W.J., Ramming, K.P., Busuttil, R.W. (1989) Effect of a prior portasystemic shunt on subsequent liver transplantation. *Ann. Surg*; **209**: 51–56.
7. Rikkers, L.F. (1988) Is the distal splenorenal shunt better? *Hepatology*; **8**: 1705–1707.
8. Rikkers, L.F., Soper, N.J., Cormier, R.A. (1984) Selective operative approach fr variceal hemorrhage. Am. J. Surg; **147**: 89–96.

### SURGERY OR ENDOPROSTHESIS FOR MALIGNANT OBSTRUCTIVE JAUNDICE

### ABSTRACT

*Shepherd HA, Royle G, Ross APR, Diba A, Arthur M and Colin-Jones D (1988) Endoscopic biliary endoprosthesis in the palliation of malignant obstruction of the distal common bile duct: a randomized trial. British Journal of Surgery; 75:1166–1168*

**A total of 52 jaundiced elderly patients who had malignant obstruction of the distal common bile duct and who required palliative biliary decompression were randomized to**

**receive either an endoscopically placed biliary endoprosthesis (10 French gauge) or conventional surgical bypass. Patients within the two treatment groups were well matched and 51 were followed until their death. Patients treated with endoprosthesis had a significantly shorter initial hospital stay than those treated surgically. In the long term, overall survival in the two groups was similar and jaundice was relieved in over 90 per cent of patients. Despite more re-admissions to hospital for those patients treated endoscopically, the total time spent in hospital still remained significantly shorter in this treatment group compared with those subjected to surgery. The endoscopically placed biliary endoprosthesis is a valuable alternative to conventional surgical bypass in the palliation of extrahepatic biliary obstruction.**

### PAPER DISCUSSION

KEYWORDS: Pancreas, carcinoma; jaundice, obstructive; endoprosthesis; surgical bypass

Amidst the plethora of published uncontrolled studies of surgical, endoscopic, and percutaneous approaches to the palliation of malignant obstruction of the distal common bile duct, Shepherd and coworkers are to be congratulated for reporting the first randomized trial comparing endoscopic biliary endoprosthesis to surgical bypass in a selected group of 52 elderly jaundiced patients[1].

The recent trend exploring the use of nonoperative techniques may largely be attributed to the generally disappointing results of either surgical resection or bypass for adenocarcinoma of the head of the pancreas, and early nonrandomized reports have suggested that transhepatic percutaneous or endoscopic intubation techniques may offer equivalent relief of jaundice with comparable survival rates[2,3,4,5]. The prospective controlled trial reported by the Capetown group demonstrated no significant difference between the palliation afforded by surgical bypass or transhepatically-introduced endoprosthesis[6]. It has been suggested previously, based on the randomized trial from Middlesex and London Hospitals, that the endoscopic approach may be superior to the transhepatic route for distal common bile duct lesions in terms of relief of jaundice and 30-day mortality[7]. In this context, a controlled comparison between endoscopic and surgical methods of palliation has been eagerly anticipated. Although the report of Shepherd strongly supports the continued use of endoscopic stenting for high-risk elderly patients, a note of caution is perhaps in order, lest the continued important place of traditional surgical techniques be lost underneath a wave of enthusiasm for nonoperative approaches.

Cytologic or biopsy confirmation of adenocarcinoma of the pancreas should be obtained before the possibility of resectional therapy be denied to a good-risk patient. Five year survival rates after Whipple pancreaticoduodenectomy for pancreatic cancer are dismal, generally less than 10%[8,9,10,11], and many centers have therefore abandoned hope of curative resection for this lesion except in highly selected patients. Such pessimism, however, is not warranted for other malignant lesions of the pancreatic head may cause obstruction of the distal common bile duct. For example, five year survival after resection for distal bile duct cancer is approximately 20% and may be as high as 50% for adenocarcinoma of the ampulla[8,9,12]. The operative morbidity and mortality of pancreaticoduodenectomy is no longer prohibitive in experienced hands. The various possible tumor types producing malignant obstruction cannot always be differentiated with confidence on radiologic grounds alone, and while fine-needle aspiration cytology appears to represent an important advance in diagnostic ability, it is still often inconclusive. Open biopsy therefore continues to be required as a diagnostic modality, and, in any event, the resectability of a given pancreatic head lesion is most accurately determined at surgical exploration. It remains incumbent on the gastroenterologist or surgeon to exclude with certainty the presence of more favorable lesions amenable to potentially curative resection before embarking on a purely palliative approach.

Criteria for patient selection in controlled trials must be carefully defined, and it is important to note that in the report by Shepherd, the presence of duodenal encroachment, portal venous compromise, disseminated malignancy, carcinoma of the papilla, and failed ERCP were appropriately considered grounds for exclusion. According to the presented data, 20 of 72 patients (28%) referred for investigation were excluded (18 patients who received nonrandomized treatment were also omitted from consideration). Because the protocol allowed for crossover between groups, four patients who failed endoscopic placement and two surgical failures were managed by the alternative technique. While technical success was achieved in a respectable 82% of endoscopically treated patients, it is surprising that there were two patients in the surgical group in whom anatomic considerations were reported to preclude the construction of a satisfactory anastomosis. In our experience at Hammersmith Hospital and the University of Berne, failure to achieve an adequate biliary bypass is rare; thorough knowledge of hepatobiliary anatomy almost always allows sufficient exposure of the common hepatic or extrahepatic left hepatic duct, or more rarely a segmental bile duct exposed via the umbilical fissure in those unusual cases where tumor involves the supraduodenal common bile duct or gallbladder. In addition, the choice of surgical bypass performed in this report and others may be criticized. Many experienced hepatobiliary surgeons now prefer Roux-en-Y choledocho-jejunostomy or hepaticojejunostomy for biliary-enteric anastomosis to reconstruction utilizing the gallbladder[13], which was chosen in over half of the surgically managed cases in this series. A surgical complication rate greater than 50% seems excessive, although there was no statistical difference in complication rates between the surgical and endoscopic groups. It is interesting to note that no patient in the stented group developed gastric outlet obstruction prior to death. The additional morbidity involved in gastro-jejunostomy is fairly negligible, but the value of routine gastric bypass has long remained a controversial point among surgeons[14,15].

The inevitable difficulty inherent in interpretation of studies asserting that an alternative therapeutic modality is "as good as" conventional techniques is the ubiquitous type II statistical error, and it is disappointing that the relatively small number of patients reported in the present series demonstrated few points of difference between the two study groups. This problem is usually unavoidable in trials examining treatment of uncommon lesions, but it may be hoped that as experience with nonoperative approaches to malignant bile duct obstruction accumulates, statistical significance will emerge. The current report consists of only a three year study period.

One important point of statistical significance did emerge, however. Total hospital days for the endoscopically managed group, including total days of readmissions, was lower than the surgically treated group. However, it may equally convincingly be argued that, in terms of quality of palliation, fewer total hospital admissions and freedom from cholangitis may be a more desirable endpoint. Indeed, there was a trend favoring fewer readmissions for the surgically managed patients (13 readmissions for cholangitis in 23 stented patients compared to 3 for 25 operated patients).

Despite the use of large-bore prostheses, the problem of late cholangitis has not been eliminated. Although planned routine stent replacement may minimize the morbidity of this complication, many surgeons argue that the invariably high incidence of endoprosthesis occlusion and cholangitis weighs strongly against routine use of the endoscopic option for routine palliative purposes and that this approach should be reserved for only the most debilitated and elderly patients. While significant surgical morbidity in jaundiced, nutritionally-depleted patients appears similarly unavoidable, with meticulous operative technique and proper choice of procedure, the early complication rate should probably not exceed 30%, and late complications should be unusual. It must also be recognized that both endoscopic and percutaneous approaches also carry an approximately 30% periprocedural complication rate even in expert hands.

Surgical and endoscopic methods of palliation all offer at least temporary relief of jaundice in over 90% of patients, and therefore a nihilistic approach to palliation appears unjustified. Neither approach can be argued to yield superior longterm survival in available controlled or uncontrolled reports. Therefore the choice of palliative approach to the management of malignant obstruction of the distal common bile duct in elderly jaundiced patients must remain individualized, dictated by available local expertise and the preferences of well-informed patients and clinicians.

Jeffrey B Matthews MD
Leslie H Blumgart MD FRCS(Edin, Glas, Engl)
Department of Visceral and Transplantation Surgery
University of Berne, Inselspital
3010 BERNE
Switzerland

### REFERENCES

1. Shepherd HA, Royle G, Ross APR, Diba A, Arthur M, Colin-Jones D. (1988) Endoscopic biliary endoprosthesis in the palliation of malignant obstruction of the distal common bile duct: a randomized trial. *British Journal of Surgery*, **75**, 1166–1168.
2. Sonnenfeld T, Gabrielson N, Granqvist S, Perbeck L. (1986) Nonresectable malignant bile duct obstruction: surgical bypass or endoprosthesis. *Acta Chirurgica Scandinavica*, **152**, 297–300.
3. Dooley JS, Dick R, George P, Kirk RM, Hobbs KEF, Sherlock S. (1984) Percutaneous endoprosthesis for bile duct obstruction: complications and results. *Gastroenterology*, **86**, 905–909.
4. Leung JWC, Emery R, Cotton PB, Russell RCG, Vallon AG, Mason RR. (1983) Management of malignant obstructive jaundice at the Middlesex Hospital. *British Journal of Surgery*, **70**, 584–586.
5. Huibregtse K, Tytgat GN. (1982) Palliative treatment of obstructive jaundice by transpapillary introduction of a large-bore bile duct endoprosthesis. *Gut*, **23**, 371–375.
6. Bornman PC, Harries-Jones EP, Tobias R, Van Steigmann G, Terblanche J. (1986) Prospective controlled trial of transhepatic biliary endoprosthesis versus bypass surgery for incurable carcinoma of head of pancreas. *Lancet*, **i**, 69–71.
7. Speer AG, Cotton PB, Russell RCG, Mason RR, Hatfield ARW, Leung JWC, Macrae KD, Houghton J Lennon CA. (1987) Randomised trial of endoscopic versus percutaneous stent insertion in malignant obstructive jaundice. *Lancet*, **ii**, 57–62.
8. Lerut JP, Gianello PR, Otte JB, Kestens PJ. (1983) Pancreaticoduodenal resection: surgical experience and evaluation of risk factors in 103 patients. *Annals of Surgery*, **199**, 432–437.

9. Herter FP, Cooperman AM, Ahlborn TN, Antinori C. (1982). Surgical experience with pancreatic and periampullary cancer. *Annals of Surgery*, **195**, 274–281.
10. Connolly MM, Dawson PJ, Michelassi F. (1987) Survival in 1001 patients with carcinoma of the pancreas. *Annals of Surgery*, **206**, 366–373.
11. Warshaw AL, Swanson RS. (1988). Pancreatic cancer in 1988: possibilities and probabilities. *Annals of Surgery*, **208**, 541–553.
12. Alexander F, Rossi RL, O'Bryan M, Kettriy U, Braasch JW, Watkins E (1984). Biliary carcinoma: a review of 109 cases. *American Journal of Surgery*, **147**, 503–509.
13. Kümmerle F, Rücket K. (1984). Surgical treatment of pancreatic cancer. *World Journal of Surgery*, **8**, 889–894.
14. Sarr MG, Cameron JL. (1984) Surgical palliation of unresectable carcinoma of the pancreas. *World Journal of Surgery*, **8**, 906–918.
15. Weaver DW, Wieneck RG, Bouwman DL, Walt AJ. (1987) Gastrojejunostomy: is it helpful for patients with pancreatic cancer? *Surgery*, **102**, 608–613.

### SELECTED HPB INTERNATIONAL

### INSTRUCTIONS TO AUTHORS

This section aims to provide the readers of *HPB Surgery* with a selected abstracting service with a difference. The Editor, the Editorial board of *HPB Surgery* and local colleagues will identify and select important and seminal papers in the field of hepatic, pancreatic and biliary surgery published in other major journals. Readers are also encouraged to contact the Editor of this section with suggestions on papers to be included. An authority in the field will be asked to comment in a signed short essay or editorial on the selected papers. Both the original abstract (or summary of the abstract) and the expert essay will be published.

The essay should be approximately two printed pages (four to five double type-written pages) and contain a few selected references. It is hoped that the experts will usually applaud but sometimes criticise the paper and will add relevant comment. The format will be left to the individual essayist.

The original authors will not be asked to comment on the essay, but are invited to submit comments, if they wish, for a special section entitled "Selected HPB International Correspondence". Here they will be afforded early publication. The Editor believes that in most instances there will be no need for the authors to reply.

The essay must conform to the style of *HPB Surgery* (see instructions to authors).

Readers' comments on the aims and format of the Selected HPB International will be welcomed by the section editor.

